# Cardiac Pain and Angina Pectoris

**Published:** 1897-11-13

**Authors:** S. H. Habershon

**Affiliations:** Assistant Physician and Pathologist to the Hospital


### MEDICAL PROGRESS AND HOSPITAL CLINICS.

[*The Editor will be glad to receive offers of co-operation and contributions from members of the profession. All letters should be addressed to* THE EDITOR, AT THE OFFICE, 28 & 29, SOUTHAMPTON STREET, STRAND, LONDON, W.C.]

### CARDIAC PAIN AND ANGINA PECTORIS.

A Clinical Lecture and Demonstration given at the Brompton Hospital for Consumption and Diseases of the Chest, by S. H. HABERSHON, M.D., F.R.C.P., Assistant Physician and Pathologist to the Hospital.

Pain is a subjective symptom, and though the correct appreciation of its cause and locality depends largely upon the judgment of the physician, he is much at the mercy of his patient. A lucid and correct description of the ailment is of the greatest value in aiding the diagnosis. In a hospital patient the difficulty is increased, because the average individual gives by no means an intelligible account. Pain radiating about the area of the præcordium may arise from so many causes that we must be cautious in deciding that it is due to any affection of the heart.

Professor Balfour has described some of the various causes to which pain in this region may be due, and I am able to bring to your notice several cases to illustrate these.

**Flatulent Colic.**—Of dyspepsia it is unnecessary to speak at length. It is a common occurrence to be consulted for supposed disease of heart, when the sole cause of pain in the cardiac area is flatulent colic and a distended stomach. The præcordium is the common situation for pain in the dyspeptic. I have here to-day a patient of this character, but with complete transposition of the viscera. The heart is on the right side of the chest, the stomach resonance is found in the right hypochondrium, and the liver dulness on the left side. The flatulent pain in this case is in the cardiac area, but on the right side of the chest.

**Gastralgia.**—Gastralgia and so-called cardialgia, both probably neuralgic conditions of stomach, may be more easily mistaken for true cardiac pain. They usually occur in atonic patients, often in anæmic women and children. Only a few days ago I was discussing the question with Dr. Gee *àpropos* of a case of an anæmic child, whom I sent to him some years ago. The girl suffered from severe pain in the region of the left breast, lasting for one or two hours and recurring daily. The pain was relieved by nitro-glycerine tabloids, but was considered by Dr. Gee to be a stomach, and not a cardiac, condition.

**Pleurisy.**—The second case I have to show illustrates a further cause of pain in the præcordium that may simulate angina pectoris, The patient, who is a woman aged 35, came to me a fortnight ago complaining of severe pain in the heart, so severe that she was afraid to breathe. She suffers from advanced mitral disease, with a well-marked thrill and presystolic murmur at the apex. A careful inquiry soon elicited the fact that the pain was only on drawing the breath, and ceased when the breath was held. We could hear a loud plural friction rub a little outside the left breast. Though the patient is here for your examination this afternoon, it is more than probable that the pleurisy will have disappeared.

**Intercostal Neuralgia.**—Intercostal neuralgia often affects the nerves of the lower intercostal spaces on the left side. I have seen this in anæmic women, who are very liable to neuralgic pains. The pain is far more continuous than a true cardiac affection, though it is liable to exacerbations. As a rule there is a tender spot in the course of the intercostal nerve, or the whole side of the chest may be hyperæsthetic. In connection with this cause is the intercostal pain and extreme præcordial tenderness found in some cases of cardiac disease, and said to be due to the pressure of an enlarged and hypertrophied heart upon the intercostal spaces. In one of the patients here this afternoon, a woman who is the subject of extensive mitral disease and has a violently acting heart, there has been much suffering from a continuous pain of this character associated with hyperæsthesia of several intercostal spaces. The pain has been greatly relieved by the rest and care she has obtained in the hospital. At the same time she has had several attacks recently, and for the first time, of paroxysmal pain of the nature of angina pectoris. I shall have occasion again to refer to this pain.

The intercostal pain in such cases cannot be always due to pressure on the nerves, for the symptom is not seen in the larger number of cases of hypertrophied hearts with which we meet. It may be that an adherent pericardium or some pleural adhesion at the left base exerts some traction upon the neighbouring intercostal spaces and thus produces pain.

**Constipation.**—In anæmia, as well as in cardiac patients, I have seen severe pain occurring in the left hypochondrium extending to the cardiac area from con stipation alone. In the differential diagnosis this cause should always be eliminated.

**yalgia.**—Lastly, myalgia is sometimes the cause of pain which leads to the suspicion of cardiac disease in the patient. Only a week or two ago I was sent for by a gentleman whom I found in a state of much anxiety from a pain which he thought was due to some heart affection. I found that his temperature was raised, and that the symptom had followed a railway journey, and had possibly resulted from chill caused by a draught from an open window. This muscular rheumatism was increased by movement of the chest or upper limbs, was diminished by holding the breath, and relieved by rubbing the chest walls.

### DIAGNOSIS.

In the severe form of angina pectoris we shall all agree that the recognition of the disease is not attended with difficulty; it is in the milder forms of the affection and in the early stages that the difficulty and the importance of the differential diagnosis appear. This is especially the case in one group, to which I shall refer, where the pain appears to be dependent at first upon peripheral resistance in the small arterioles. For unless relieved a continued high arterial tension will eventually lead to grave degeneration both of the cardiac muscle and the arterial walls.

One feature which distinguishes true cardiac pain from all others is its paroxysmal character. It is not continuous, though there may be a constant sense of uneasiness or oppression. The pain may be of short duration, and of moderate intensity, or it may be of great severity, amounting to agony, and lasting for several hours. The attacks of pain are accompanied frequently by a sense of constriction or suffocation, though the respiration is not necessarily affected. The patient does not, however, breathe freely or deeply, but this often appears to be because of the fear of increasing a pain which is already scarcely endurable. In some cases, however, it seems as if the pulmonary vessels shared in the general vasomotor spasm.

Paroxysms, when slight, may recur many times in the day or night, or we may find one severe attack not returning for days, or even years. In the classical and idiopathic disease to which we apply Heberden's name of angina pectoris, it is well known that the seizure may in some cases be speedily fatal, and even in a first attack death may occur, as in the oft-told and well-known case of Dr. Arnold, of Rugby.

This spasmodic pain is further characterised by a sense of dread or of impending dissolution, and this is to be distinguished from the merely nervous fear that there is some affection of the heart.

Syncope or faintness is a frequent concomitant, especially in severe attacks.

The temperature is not raised, thus distinguishing the disease from other affections accompanied by pain in the chest and of inflammatory origin.

### Causes.

The subject of angina pectoris is still one of some obscurity, and in the literature of the subject there is a good deal of difference of opinion expressed both as to the ultimate cause of the affection and as to the cases which should be included under the term. It must be remembered that the term angina pectoris is applied to a group of symptoms, and I personally deprecate its application only to that form of the disease which is associated with a degenerate heart or atheromatous coronary arteries without gross cardiac lesion, and accompanied at least during the attack by a small pulse of high tension.

Angina pectoris, or the symptoms which the term implies, may, like asthma, be produced by a variety of causes, and while reserving the term pseudo-angina, or spurious angina, for conditions simulating the classical disease, I prefer to deal with the subject on a broader basis. In the admirable paper with which Sir R. Douglas Powell opened a discussion at the Medical Society in the early part of 1891 the subject was treated in this wise.

### Nervous and Physical Relations.

We have to consider that the heart and arterial system is controlled by the vasomotor centre in the brain, that the heart alone is regulated by its inhibitory mechanism, of which the chief factor is the pneumogastric nerve, and by an accelerator system of nerves through the agency of the sympathetic. Further, that at the base of the heart are not only a series of cardiac plexuses communicating with the pneumogastric and sympathetic nerves, but also in the cardiac tissues a series of ganglia or ganglionic cells, of which the function is still somewhat obscure. Not only may the heart alone be accelerated, depressed, or thrown into a condition of spasm by means of its controlling nervous mechanism, but by a vasomotor spasm of the small arterioles the blood tension may be so raised that the cardiac movements and actions are embarrassed.

So much for the nervous mechanism; but the heart can be influenced and embarrassed by a rise of blood pressure and pulse tension, caused by peripheral obstruction not arising directly from the central nervous system. A purely peripheral cause may thus be the inducing factor of a true cardiac spasm.

Finally (and these constitute the most important and classical group of cases), a degenerative change in the cardiac tissues, and, above all, a deficient blood supply due to atheroma or obstruction of the coronary arteries, may act, as some think, directly on the cardiac muscle, producing a tonic spasm; or as others, including the French school, express it: "The cardiac nerves and ganglia are capable of transmitting an impulse to the vasomotor centre, which cause it to throw the smaller vessels into contraction."

Thus, causes may arise in the brain (vasomotor centre), the cardiac nervous mechanisms, the cardiac muscle or ganglia, and the peripheral arterial system any or all of which may produce the remarkable spasm of heart and the associated group of symptoms which are known as "breast pang," or "angina pectoris."

*(To be continued.)*

